# Hypoglycemia induced changes in cholinergic receptor expression in the cerebellum of diabetic rats

**DOI:** 10.1186/1423-0127-17-7

**Published:** 2010-02-05

**Authors:** Sherin Antony, Peeyush Kumar T, Jobin Mathew, TR Anju, CS Paulose

**Affiliations:** 1 Molecular Neurobiology and Cell Biology Unit, Centre for Neuroscience, Department of Biotechnology, Cochin University of Science and Technology, Cochin - 682 022, Kerala, India

## Abstract

Glucose homeostasis in humans is an important factor for the functioning of nervous system. Hypoglycemia and hyperglycemia is found to be associated with central and peripheral nerve system dysfunction. Changes in acetylcholine receptors have been implicated in the pathophysiology of many major diseases of the central nervous system (CNS). In the present study we showed the effects of insulin induced hypoglycemia and streptozotocin induced diabetes on the cerebellar cholinergic receptors, GLUT3 and muscle cholinergic activity. Results showed enhanced binding parameters and gene expression of Muscarinic M1, M3 receptor subtypes in cerebellum of diabetic (D) and hypoglycemic group (D + IIH and C + IIH). α7nAchR gene expression showed a significant upregulation in diabetic group and showed further upregulated expression in both D + IIH and C + IIH group. AchE expression significantly upregulated in hypoglycemic and diabetic group. ChAT showed downregulation and GLUT3 expression showed a significant upregulation in D + IIH and C + IIH and diabetic group. AchE activity enhanced in the muscle of hypoglycemic and diabetic rats. Our studies demonstrated a functional disturbance in the neuronal glucose transporter GLUT3 in the cerebellum during insulin induced hypoglycemia in diabetic rats. Altered expression of muscarinic M1, M3 and α7nAchR and increased muscle AchE activity in hypoglycemic rats in cerebellum is suggested to cause cognitive and motor dysfunction. Hypoglycemia induced changes in ChAT and AchE gene expression is suggested to cause impaired acetycholine metabolism in the cerebellum. Cerebellar dysfunction is associated with seizure generation, motor deficits and memory impairment. The results shows that cerebellar cholinergic neurotransmission is impaired during hyperglycemia and hypoglycemia and the hypoglycemia is causing more prominent imbalance in cholinergic neurotransmission which is suggested to be a cause of cerebellar dysfunction associated with hypoglycemia.

## Introduction

Hypoglycemic brain injury is a common and serious complication of insulin therapy in diabetic individuals [1,2]. Studies suggest that acute or chronic hypoglycemia leads to neurological dysfunction and injury. Severe hypoglycemia triggers a cascade of events in vulnerable neurons that culminate in cell death even after glucose normalization [3-5]. Children and adults exposed to hypoglycemia can develop long-term impairment of cognitive function [6] and are at risk of epilepsy.

Altered neurotransmitter action appears to play a role in hypoglycemic brain dysfunction [7-9]. Muscarinic acetylcholine receptors play important roles in many fundamental central functions including higher cognitive processes and modulation of extrapyramidal motor activity. Synaptic ACh levels are known to be regulated by the activity of presynaptic muscarinic autoreceptors mediating inhibition of ACh release. In terms of the contribution of cholinergic cerebellar abnormalities to mental function, early reports of cerebellar abnormalities in autism [10] and of intellectual and behavioural abnormalities in patients with cerebellar damage [11] originally suggested a cognitive role for the cerebellum. Since then, many studies have confirmed that the cerebellum contributes to cognitive and other non-motor functions. There is thus increasing evidence that the cerebellum is involved in cognition, behaviour and emotion [12]. Cerebellar dysfuncton is associated with poor fine motor skills, hypotonia [13]. Alterations in glucose utilization are known to occur in the important regions of brain connected with learning and memory [14,15].

Receptors activate a multitude of signaling pathways important for modulating neuronal excitability, synaptic plasticity and feedback regulation of ACh release [16]. In the cerebellum, nicotinic acetylcholine receptors mediate the release of glutamate [17], GABA [18,19] and norepinephrine [20]. Thus, these receptors significantly influence the activity within the cerebellar circuitry, and any deregulation of this activity contributes to functional disorders involving the cerebellum.

The altered levels of neurotransmitter in specific brain areas in patients with diabetes mellitus [21] and in animals with experimental diabetes [22-27] have been documented and implicated in the CNS disorders. Recently we have reported that muscarinic M1 receptor gene expressions were decreased in the cerebral cortex, brainstem, hypothalamus and pancreatic islets of STZ induced diabetic rats and insulin modulates the binding parameters and gene expression [28,29].

Moderate hypoglycemia is known to have significant impact on functions of the central nervous system, and any differential effect of hypoglycemia on the peripheral nervous system may offer insights into the metabolic requirements of central and peripheral neurons [30]. In a case of episodic bilateral cerebellar dysfunction caused by hypoglycemia, quantitative dynamic PET study demonstrated decreased glucose uptake-to-utilization ratio and increased leak of glucose in the cerebellum indicating that cerebellum is not invariably resistant to hypoglycemia [31]. Studies from our laboratory have demonstrated that cerebellum is susceptible to hypoglycemia [32,33]. Studies on damages of the central nervous system under conditions of hypoglycemia are very important for clinical medicine. The main objective of the present study was to determine whether hypoglycemia as a consequence of insulin therapy in diabetes altered the binding parameters of Muscarinic M1, M3 receptors and gene expression of α7nAchR, AchE, ChAT and GLUT3 in the cerebellum and AchE activity in the muscle of experimental rats.

## Materials and methods

Male adult Wistar rats of 200-250 g body weight were used for all experiments. Animals were divided into the following groups as (i) control (C), (ii) diabetic (D), (iii) insulin-induced hypoglycemia in diabetic rats (diabetic + IIH) and (iv) insulin-induced hypoglycemia in control rats (control + IIH). Each group consisted of 6-8 rats. They were housed in separate cages under 12-h light and 12-h dark periods and were maintained on standard food pellets and water *ad libitum*. All animal care and procedures were in accordance with Institutional and National Institute of Health guidelines.

Diabetes was induced in rats by single intrafemoral injection of streptozotocin (Sigma Chemical Co., St. Louis, MO) freshly dissolved in 0.1 M citrate buffer, pH 4.5, under anesthesia. Streptozotocin was given at a dose of 55 mg/kg body weight [34,35]. The diabetic + IIH group received daily 2 doses (10 Unit/kg body weight) and control + IIH group received daily 2 doses (1.5 Unit/kg body weight) of regular human insulin (Actrapid) [36]. Diabetic + IIH and control + IIH group had daily two episodes of insulin-induced hypoglycemia for 10 days. Control rats were injected with citrate buffer. Glucose was measured by GOD-POD glucose estimation kit (Biolab Diagnostics Pvt Ltd). Rats were sacrificed by decapitation on the 10^th ^day of the experiment. The cerebellum was dissected out quickly over ice according to the procedure of Glowinski and Iversen [37] and the tissues collected were stored at -80°C until assayed.

### Muscarinic M1 and M3 Receptor Binding Studies in the Cerebellum

Muscarinic M1 and M3 receptor binding assays were done using specific antagonists [^3^H]QNB and [^3^H]DAMP in the cerebellum of rat groups respectively [38]. The tissues were homogenised in a polytron homogeniser with 20 volumes of cold 50 mM Tris-HCl buffer, pH 7.4 containing 1 mM EDTA. The supernatant was then centrifuged at 30,000 × g for 30 minutes and the pellets were suspended in appropriate volume of Tris-HCl- EDTA buffer. Muscarinic M1 binding assay was done using different concentrations i.e., 0.1-2.5 nM of [^3^H] QNB in the incubation buffer, pH 7.4 in a total incubation volume of 250 μl containing appropriate protein concentrations (200-250 μg). Nonspecific binding for muscarinic M1 receptor was determined using 100 μM of pirenzepine (Sigma Chemical Co.). Muscarinic M3 binding assay was done using different concentrations i.e., 0.1-2.5 nM of [^3^H] DAMP in the incubation buffer, pH 7.4 in a total incubation volume of 250 μl containing appropriate protein concentrations (200- 250 μg). Nonspecific binding for muscarinic M3 receptor was determined using 100 μM of 4-DAMP mustard.

Tubes were incubated at 22°C for 60 minutes and filtered rapidly through GF/C filters (Whatman). The filters were washed quickly by three successive washing with 5.0 ml of ice cold 50 mM Tris-HCl buffer pH 7.4. Bound radioactivity was counted with cocktail-T in a Wallac 1409 liquid scintillation counter.

### Analysis of gene expression by Real-time PCR

RNA was isolated from the cerebellum using Tri reagent. Total cDNA synthesis was performed using ABI PRISM cDNA Archive kit. Real-Time PCR assays were performed in 96-well plates in ABI 7300 Real-Time PCR instrument (Applied Biosystems). PCR analyses were conducted with gene-specific primers and fluorescently labeled Taq for Muscarinic M1, M3, α7nAchR, ChAT, AchE and GLUT3 mRNA (designed by Applied Biosystems). Endogenous control, β-actin was labeled with a report dye (VIC). All reagents were purchased from Applied Biosystems.

The thermocycling profile conditions were as follows: 50°C for 2 min -Activation, 95°C for 10 min - Initial Denaturation, 95°C for 15 s - Denaturation 40 cycles, 50°C for 30 s - Annealing, 60°C for 1 min - Final Extension. The ΔΔCT method of relative quantification was used to determine the fold change in expression. This was done by first normalizing the resulting threshold cycle (CT) of the target mRNAs to the CT-values of the internal control β-actin in the same samples (ΔCT = CT Target - CT β-actin). It was further normalized with the control (ΔΔCT = ΔCT - CT Control). The fold change in expression was then obtained (2^-ΔΔCT^).

### Acetylcholine Esterase Assay in the muscle of control and experimental rats

Acetylcholine esterase assay was done using the spectrophotometric method of Ellman et al [39]. The homogenate (10%) was prepared in 30 mM sodium phosphate buffer, pH 8.0. One ml of 1% Triton X 100 was added to the homogenate to release the membrane bound enzyme and centrifuged at 12,500 × g for 30 minutes at 4°C. Different concentrations of acetylthiocholine iodide were used as substrate. The mercaptan formed as a result of the hydrolysis of the ester reacting with an oxidising agent 5,5' -dithiobis (2-Nitrobenzoate) was read at 412 nm.

### Protein Determination

Protein was measured by the method of Lowry et al [40] using bovine serum albumin as standard.

### Statistical Analysis

Statistical evaluations were done with analysis of variance (ANOVA), using GraphPad Instat (version 2.04a, San Diego, USA).

## Results

### Blood glucose level in diabetic, Diabetic + IIH and Control + IIH Rats

Blood glucose level of all rats before streptozotocin administration and control rats during the treatment period was within the normal range (80-105 mg/dl). Streptozotocin administration to rats brought about significant (P < 0.001) increase in blood glucose level when compared to control (Table 1). The insulin induced hypoglycemic group showed a significant (P < 0.001) reduction in blood glucose level.

**Table 1:** Blood glucose levels of Control, Diabetic, Diabetic + IIH and Control + IIH rats

Animal Status	Blood Glucose (mg/dL)
Control	108 ± 5.77

Diabetic	257 ± 3.18^a^

D+IIH	47 ± 3.05^a b^

C+IIH	44 ± 1.45^a b^

Values are Mean ± S.E.M. of 4-6 separate experiments. Each group consists of 6-8 rats.

**a **p < 0.001 when compared to control, **b **p < 0.001 when compared to Diabetic (D).

IIH- Insulin Induced Hypoglycemia.

### Enhanced Muscarinic M1, M3 receptor binding in the Cerebellum of Diabetic, Diabetic + IIH and Control + IIH Rats

Scatchard analysis of [^3^H] QNB Binding against Pirenzepine to study muscarinic M1 receptor binding parameters showed a significant increase in B_max _in the cerebellum of hypoglycemic (P < 0.001) and diabetic (P < 0.001) rats when compared to control. Diabetic hypoglycemic and control hypoglycemic group showed a significant increase (P < 0.001) in B_max _compared to diabetic rats. Control hypoglycemic group showed a significant increase (P < 0.001) when compared to Diabetic hypoglycemic group. The K_d _value of both diabetic hypoglycemic and Control hypoglycemic groups showed an increase (P < 0.01) when compared to control and diabetic group (Table 2).

**Table 2:** Scatchard analysis of [^3^H] QNB binding against pirenzepine in the cerebellum of Control, Diabetic, Diabetic + IIH and Control + IIH Group of rats

Experimental Group	Bmax (fmoles/mg protein)	Kd (nM)
Control	127 ± 12.4	0.40 ± 0.03

Diabetic	183 ± 11.5 ^**a**^	0.41 ± 0.02

D + IIH	245 ± 10.5 ^**ab**^	0.50 ± 0.03^**d**^

C + IIH	296 ± 9.8 ^**abc**^	0.54 ± 0.02^**d**^

Values are Mean ± S.E.M. of 4-6 separate experiments. Each group consists of 6-8 rats. a p < 0.001 when compared to control, b p < 0.001 when compared to Diabetic, c p < 0.01 when compared to D + IIH, d p < 0.01 when compared to diabetic. IIH- Insulin Induced Hypoglycemia.

Scatchard analysis of [3H] DAMP Binding against 4- DAMP parameters showed to study muscarinic M3 receptor binding showed a significant increase in B_max _in the cerebellum of hypoglycemic (P < 0.01) and diabetic (P < 0.001) rats when compared to control. Diabetic hypoglycemic and control hypoglycemic group showed a significant increase (P < 0.01) in B_max _compared to diabetic rats. Kd of Control hypoglycemic group showed a significant decrease (P < 0.01) when compared to diabetic and diabetic hypoglycemic group (Table 3).

**Table 3:** Scatchard analysis of [^3^H] DAMP binding against 4 DAMP in the cerebellum of Control, Diabetic, Diabetic + IIH and Control + IIH Group of rats

Experimental Group	Bmax (fmoles/mg protein)	Kd (nM)
Control	12 ± 1.5	0.46 ± 0.02

Diabetic	17 ± 0.5 ^**a**^	0.45 ± 0.02

D + IIH	20 ± 0.4 ^**ab**^	0.43 ± 0.01

C + IIH	22 ± 0.5 ^**ab**^	0.36 ± 0.01^**c**^

Values are Mean ± S.E.M. of 4-6 separate experiments. Each group consists of 6-8 rats. a p < 0.01 when compared to control, b p < 0.01 when compared to Diabetic, c p < 0.01 when compared to C, D, D + IIH, IIH- Insulin Induced Hypoglycemia.

### Increased AchE activity in the muscle of Diabetic, Diabetic + IIH and Control + IIH Rat

AchE activity in the muscle showed a significant increase (p < 0.001) in insulin induced hypoglycemia in both diabetic and control rats when compared to control and significant increase (p < 0.01) when compared to diabetic rats (Table 4).

**Table 4:** Acetylcholine esterase activity in the muscle of Control and experimental rats

Animal status	Vmax (Enzyme Units/mg ptn)	Km (mM)
Control	8.40 ± 0.02	0.20 ± 0.04

Diabetic	6.58 ± 0.07 ^**a**^	0.22 ± 0.06

D + IIH	16.00 ± 0.06 ^**ab**^	0.20 ±0.04

C + IIH	15.15 ± 0.08 ^**ab**^	0.20 ± 0.10

Values are Mean ± S.E.M. of 4-6 separate experiments. Each group consists of 6-8 rats. a p < 0.001 when compared to control, b p < 0.01 when compared to Diabetic. IIH- Insulin Induced Hypoglycemia.

### Up regulation of Muscarinic M1, M3, α7nAchR, AchE, GLUT3 mRNA and down regulation of ChAT gene expression in cerebellum of Diabetic, Diabetic + IIH and Control + IIH Rat

**Figure 1:**
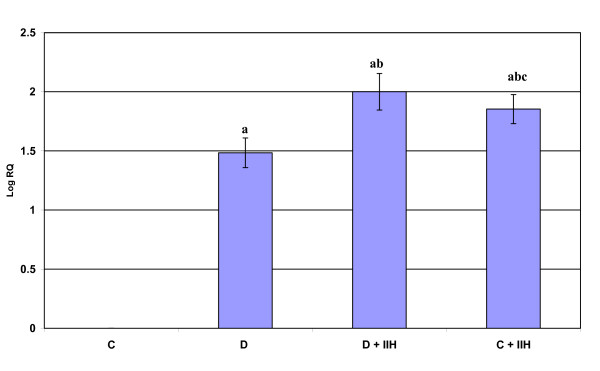
**Representative graph showing Real Time PCR amplification of muscarinic M1 mRNA from the cerebellum of Control, Diabetic, Diabetic + IIH and Control + IIH Rats**. The ΔΔCT method of relative quantification was used to determine the fold change in expression with β-actin CT value as the internal control and Control CT value as the calibrator. C- Control, D- Diabetic, D + IIH - Insulin induced hypoglycemia in diabetic, C + IIH - Insulin induced hypoglycemia in control. Values are mean ± S.D. of 4-6 separate experiments. Each group consisted of 6-8 rats. a p < 0.001 when compared to control, b p < 0.01 when compared to Diabetic, c p < 0.001 when compared to D + IIH.

**Figure 2:**
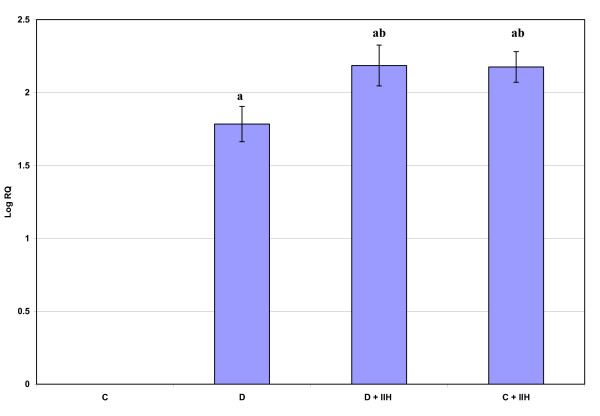
**Representative graph showing Real Time PCR amplification of muscarinic M3 mRNA from the cerebellum of Control, Diabetic, Diabetic + IIH and Control + IIH Rats**. The ΔΔCT method of relative quantification was used to determine the fold change in expression with β-actin CT value as the internal control and Control CT value as the calibrator. C- Control, D- Diabetic, D + IIH - Insulin induced hypoglycemia in diabetic, C + IIH - Insulin induced hypoglycemia in control. Values are mean ± S.D. of 4-6 separate experiments. Each group consisted of 6-8 rats. a p < 0.001 when compared to control. b p < 0.001 when compared to Diabetic.

Real-time PCR analysis of Muscarinic M1 receptor mRNA showed a significant up regulation (p < 0.001) in diabetic and hypoglycemic rats when compared to control. Diabetic hypoglycemic and control hypoglycemic group showed a significant up regulation (p < 0.01) when compared to diabetic group. Control hypoglycemic showed a significant up regulation (p < 0.001) when compared to diabetic hypoglycemic group. (Fig: 1). Real-time PCR analysis of Muscarinic M3 receptor mRNA showed a significant up regulation (p < 0.001) in diabetic and hypoglycemic rats when compared to control. Diabetic hypoglycemic and control hypoglycemic group showed a significant up regulation (p < 0.001) when compared to diabetic group (Fig: 2)

**Figure 3:**
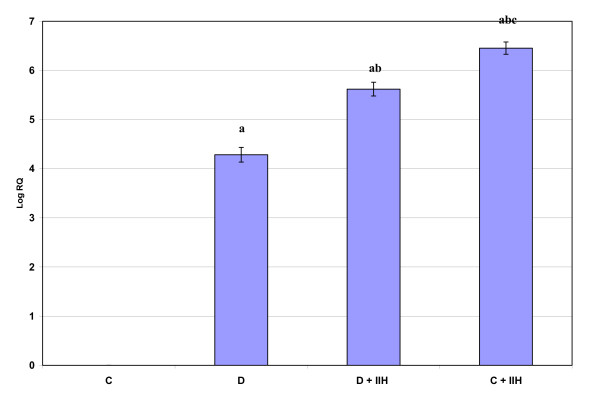
**Representative graph showing Real Time PCR amplification of α7nAchR mRNA from the cerebellum of Control, Diabetic, Diabetic + IIH and Control + IIH Rats**. The ΔΔCT method of relative quantification was used to determine the fold change in expression with β-actin CT value as the internal control and Control CT value as the calibrator. C- Control, D- Diabetic, D + IIH - Insulin induced hypoglycemia in diabetic, C + IIH - Insulin induced hypoglycaemia in control. Values are mean ± S.D. of 4-6 separate experiments. Each group consisted of 6-8 rats. a p < 0.001 when compared to control, b p < 0.001 when compared to Diabetic, c p < 0.001 when compared to D + IIH.

α7nAchR mRNA expression showed a significant (P < 0.001) up regulation in diabetic rats when compared to control. The diabetic hypoglycemic and control hypoglycemic rats showed a significant up regulation (P < 0.001) when compared to control. Control hypoglycemic group showed a significant increase (P < 0.001) when compared to diabetic hypoglycemic groups. (Fig: 3).

**Figure 4:**
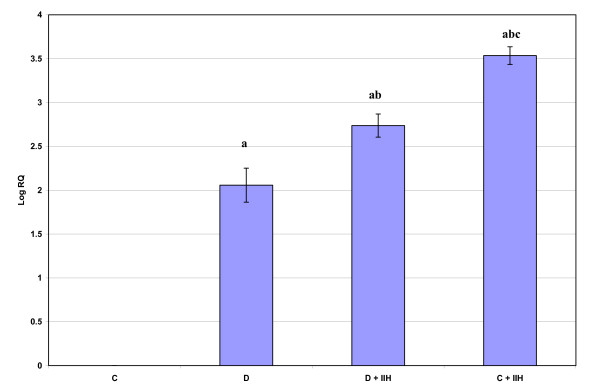
**Representative graph showing Real Time PCR amplification of AchE mRNA from the cerebellum of Control, Diabetic, Diabetic + IIH and Control + IIH Rats**. The ΔΔCT method of relative quantification was used to determine the fold change in expression with β-actin CT value as the internal control and Control CT value as the calibrator. C- Control, D- Diabetic, D + IIH - Insulin induced hypoglycemia in diabetic, C + IIH - Insulin induced hypoglycemia in control. Values are mean ± S.D. of 4-6 separate experiments. Each group consisted of 6-8 rats. a p < 0.001 when compared to control, b p < 0.001 when compared to Diabetic, c p < 0.001 when compared to D + IIH.

AchE mRNA expression showed an increased gene expression (P < 0.001) in diabetic and hypoglycemic group when compared to control. The diabetic hypoglycemic and control hypoglycemic rat group showed an increased gene expression (P < 0.001) when compared to diabetic group. Control hypoglycemic group showed a significant increase (P < 0.001) when compared to diabetic hypoglycemic groups. (Fig: 4).

**Figure 5:**
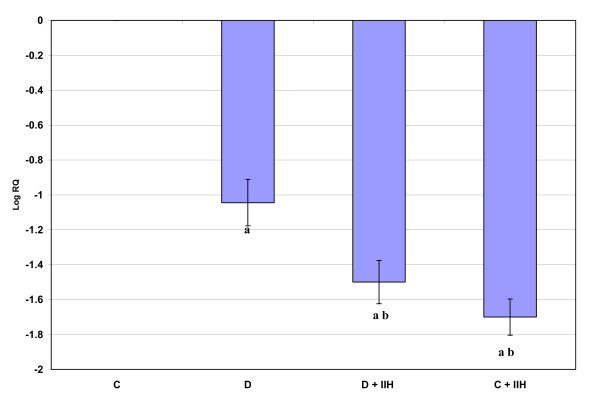
**Representative graph showing Real Time PCR amplification of ChAT mRNA from the cerebellum of Control, Diabetic, Diabetic + IIH and Control + IIH Rats**. The ΔΔCT method of relative quantification was used to determine the fold change in expression with β-actin CT value as the internal control and Control CT value as the calibrator. Values are mean ± S.D. of 4-6 separate experiments. Each group consisted of 6-8 rats. a p < 0.001 when compared to control, b p < 0.001 when compared to Diabetic.

ChAT expression showed a significant decrease (P < 0.001) in diabetic rats when compared to control. The diabetic hypoglycemic and control hypoglycemic rats showed a significant downregulation (P < 0.001) when compared to control. (Fig: 5).

**Figure 6:**
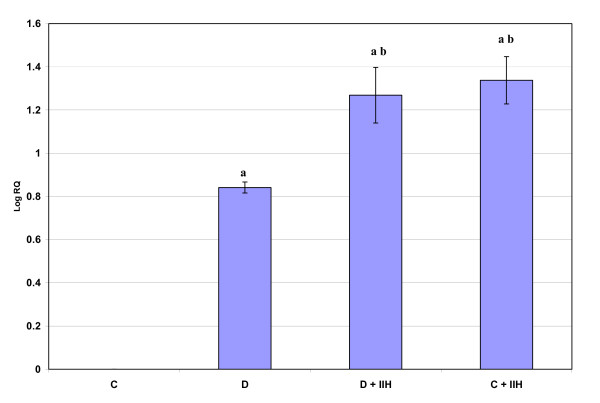
**Representative graph showing Real Time PCR amplification of GLUT3 mRNA in the cerebellum of Control, Diabetic, Diabetic + IIH and Control + IIH Rats**. The ΔΔCT method of relative quantification was used to determine the fold change in expression with β-actin CT value as the internal control and Control CT value as the calibrator. C- Control, D- Diabetic, D + IIH - Insulin induced hypoglycemia in diabetic, C + IIH - Insulin induced hypoglycemia in control. Values are mean ± S.D. of 4-6 separate experiments. Each group consisted of 6-8 rats. a p < 0.001 when compared to control, b p < 0.001 when compared to Diabetic.

GLUT3 mRNA in the cerebellum showed a significant up regulation in gene expression (P < 0.001) in diabetic rats and hypoglycemic group when compared to control. The diabetic hypoglycemic and control hypoglycemic rats also showed a significant increased (P < 0.001) gene expression compared to diabetic (Fig: 6).

## Discussion

Hypoglycemia impose alterations upon both the central (CNS) and peripheral (PNS) nervous systems which leads to brain damage and long-term cognitive impairment. The brain and other tissues require glucose in order to function properly. Neurotransmitters show significant alterations during hyperglycemia and cause degenerative changes in neurons of the central nervous system [41,42]. Severe hypoglycemia with brain dysfunction limits intensified therapy in patients with insulin dependent diabetes mellitus, despite evidence that such therapy reduces the risk of chronic complications of the disease [43].

In our earlier studies, we reported the glutamate mediated excitotoxicity in the cerebellum of insulin induced hypoglycemic and streptozotocin induced diabetic rats [32]. In the present study, we have demonstrated the role of cholinergic receptors during recurrent hypoglycemia in diabetic rats. Experimental evidence indicate the involvement of the cerebellum in variety of human mental activities including language, attention, cognitive affective syndromes [44] and motor relearning [45]. The cerebellar vermis integrates and processes the inputs from the vestibular, visual and proprioceptive systems to coordinate muscle timing as a result of which the centre of gravity stays within the limits of stable upright standing [46]. Cerebellum participates in learning and coordination of anticipatory operations which are necessary for the effective and timely directing of cognitive and non-cognitive resources [47]. Diabetes mellitus has been reported to be accompanied by a number of behavioral and hormonal abnormalities, including reduced locomotor activity [48]. Acute hypo- and hyperglycemia have disruptive effects on the central nervous system [49,50]. Complications associated with diabetes involve neuronal damage which leads to altered neurotransmitter functions and reduced motor activity.

Glucose sensitive neurons organize and respond to changes in a number of hormonal, metabolic, transmitter, and peptide signals which involve the regulation of energy homeostasis and other biological functions [51]. Glucose deprivation causes neuronal death affecting the cognitive and memory ability. Hypoglycemia and glucose deprivation causes mitochondrial damage [52]. GLUT3 is one of the predominant glucose transporters located on neurons [53]. GLUT3 had its highest expression in brain and neural tissue hence being called the brain glucose transporter [54]. Our results shows an increased gene expression of GLUT3 expression in cerebellum of diabetic group and the hypoglycemic group showed a significant increase compared to diabetic group which shows that cerebellar glucose transport impairment is maximal during insulin induced hypoglycemia leading to neuronal dysfunction. Recent study demonstrated decreased glucose uptake-to-utilization ratio and increased leak of glucose in the cerebellum which showed that the cerebellum is not invariably resistant to hypoglycemia [55]. Disorders in the transport and metabolism of glucose are an important signal for triggering the apoptotic cascade [56].

Changes in acetylcholine receptor have been implicated in the pathophysiology of many major diseases of the central nervous system. As in brain injury associated with ischaemia and neurodegenerative conditions, altered neurotransmitter action appears to play a role in hypoglycemic brain injury [7-9]. Cholinergic receptors activate a multitude of signaling pathways important for modulating neuronal excitability, synaptic plasticity and feedback regulation of ACh release [16]. The Muscarinic acetylcholine receptors are widely distributed throughout the body, but are predominantly expressed within the parasympathetic nervous system and exert both excitatory and inhibitory control over central and peripheral tissues. In the present study, enhanced muscarinic M1 and M3 receptor binding in the cerebellum of insulin induced hypoglycemia in both diabetic and nondiabetic rats along with increased AchE activity and decreased ChAT expression shows altered acetylcholine metabolism in the cerebellum. Cognitive deficits are reported to be connected with impairments of the cholinergic system [57]. Muscarinic acetylcholine receptor subtypes together with the activity of the cholinesterases (ChEs), mediate facilitation or depression of synaptic transmission [58] and AChE activity has been found to determine the range of ACh concentrations. Previous reports shows that insulin-induced hypoglycemia in normothermic rats caused progressive neurological depression and differentially altered regional cerebral acetylcholine metabolism [59].

Neuronal nicotinic cholinergic receptors are crucial to acetylcholine neurotransmission in CNS. Our results show a significant upregulation in α7nAchR gene expression induced by hypoglycemia in diabetes and control rats when compared to diabetic rats which is suggested to cause nicotinic receptor mediated dysfunction. α7nAChRs are located in brain areas important for cognition and dysfunction of α7nAChRs in cerebellum is associated with cholinergic deficit. In the cerebellum, nicotinic acetylcholine receptors mediate the release of glutamate [17], GABA [18] and norepinephrine [20]. Thus, these receptors significantly influence the activity within the cerebellar circuitry, and any deregulation of this activity contributes to functional deficit.

Acetylcholine mediated neurotransmission is involved in neuromuscular functions cerebellar dysfunction is associated with poor fine motor skills and hypotonia [13]. Acetylcholinesterase is critical for ensuring normal synaptic transmission. It is found that patients who recover from severe hypoglycemia are left with difficulties in cognition, particularly short-term memory, out of proportion to gross motor disability [4]. Our results showed an increased acetylcholine esterase activity in the muscle of hypoglycemic rats compared to diabetic group which shows neuromuscular dysfunction mediated by acetylcholine in the muscle of experimental rats. Up regulation of glutamate receptor activity causing motor dysfunction associated with cerebellum was demonstrated by the rotarod test in our previous studies [60]. Integrity of the neuromuscular junction is altered during hypoglycemia as reported by Thomareis et al [61]. It is observed that there is occurrence of seizures in hypoglycemic state which is due to the decreased glucose for the brain cells to function [62].

To summarise, our results shows dysfunction of cerebellar cholinergic receptor due to impaired neuronal glucose transport in the cerebellum during recurrent hypoglycemia in diabetic rats. The receptor analysis and gene expression studies along with muscle acetylcholine esterase activity implicate a role for acetylcholine and cholinergic receptors in the modulation of neuronal network excitability and neuromuscular dysfunction associated with hypoglycemia. Our results supports previous reports that cerebellum is not spared during recurrent hypoglycemia in diabetes. These neurofunctional deficits are one of the key contributors to motor deficits and cellular stress associated with hypoglycemia in diabetes which is suggested to cause more damage at molecular level than hyperglycemia.

## Abbreviations

AchE: acetycholine esterase; ChAT: choline acetyltransferase; α7nAchR: alpha7 nicotinic acetylcholine receptor; QNB: Quinuclidinyl benzilate; L: benzilic - 4,4'; DAMP: 4- deoxy acetyl methyl piperidine mustard; D + IIH: Insulin induced hypoglycemia in diabetes; C + IIH: Insulin induced hypoglycemia in Control.

## Competing interests

The authors declare that they have no competing interests.

## Authors' contributions

SA and CSP designed research. SA and PKT carried out experiments and drafted the manuscript. JM and ATR helped in experiments. All authors read and approved the final manuscript.
